# The relationship between coaching leadership behaviors and athletes’ psychological fatigue: the mediating role of psychological resilience

**DOI:** 10.3389/fpsyg.2026.1828916

**Published:** 2026-07-01

**Authors:** Hongfei Wang, Haozhan Gao, Chunyu Yang, Lunan Zhao

**Affiliations:** 1 College of Physical Education and Sports Science, Qufu Normal University, Qufu, Shandong Province, China; 2 School of Physical Education, Keimyung University, Daegu, Republic of Korea; 3 Moray House School of Education and Sport, The University of Edinburgh, Edinburgh, United Kingdom

**Keywords:** athletes’ psychological fatigue, basketball player, coaching leadership behaviors, psychological resilience, sports mental health

## Abstract

**Objective:**

This study aims to explore the relationship between coaches’ behaviors and athletes’ psychological fatigue, and to examine the mediating role of psychological resilience in this relationship.

**Methods:**

Using convenience sampling, a questionnaire survey was conducted with 332 Chinese basketball players from Shandong, Sichuan, Guangdong, Zhejiang, and Liaoning provinces (63.3% male, 36.7% female; with 61.1% aged 18–21). The assessment tools included the Coach Leadership Behavior Scale, the Sport Mental Toughness Questionnaire, and the Sport Psychological Fatigue Scale.

**Results:**

Correlation analysis indicated significant correlations among the study variables. Regression analysis further revealed that authoritarian leadership behavior positively predicted psychological fatigue (*β* = 0.30, *p* < 0.001), while coaching (*β* = −0.21, *p* < 0.001), democratic behavior (*β* = −0.14, *p* < 0.05), social support (*β* = −0.19, *p* < 0.01), and positive feedback (*β* = −0.16, *p* < 0.01) negatively predict psychological fatigue. Mediational analysis indicated that psychological resilience partially mediated the relationship between the five dimensions of coaching leadership behavior and psychological fatigue, with indirect effects accounting for 38.18 to 88.05% of the total effect.

**Conclusion:**

This study reveals significant correlations among coaching leadership behaviors, athletic psychological resilience, and athletes’ psychological fatigue, as well as among these dimensions themselves. Coaching leadership behaviors directly influence athletes’ psychological fatigue and indirectly affect it through the mediating role of psychological resilience.

## 1 Introduction

As the level of athletic competition continues to rise, alleviating athletes’ psychological fatigue has become a crucial factor in maintaining their performance and enhancing their results (McCormick et al., 2012; Pageaux and Lepers, 2018). Athletes’ psychological fatigue is a psychological syndrome caused by prolonged training and competition stress, characterized by emotional/physical exhaustion, diminished sense of accomplishment, and negative evaluations of the sport (Gustafsson et al., 2011). Athletes’ psychological fatigue not only harms athletes’ physical and mental health, but may also lead to the premature end of their athletic careers (Gustafsson et al., 2017). Research indicates that athletes experiencing prolonged mental fatigue exhibit significant cognitive impairments, such as reduced decision-making accuracy and decreased visual search efficiency (Fortes et al., 2020). In basketball specifically, mental fatigue has been shown to impair technical performance, including free-throw shooting accuracy, three-point shooting, and passing precision during gameplay (Filipas et al., 2021; Moreira et al., 2018). On the physical level, this results in diminished endurance performance and total running distance due to an increased subjective perception of exertion (Chaudhuri and Behan, 2004). Given that coaches, as the primary architects of the daily training environment, hold a position of high authority in athletes’ psychosocial adaptation (Callow et al., 2009; Vella et al., 2013). Investigating how coaching leadership behaviors influence athletes’ psychological fatigue holds significant practical importance for designing evidence-based coach education programs and targeted interventions aimed at safeguarding athletes’ mental health and sustaining their long-term competitive engagement.

**Figure 1:**
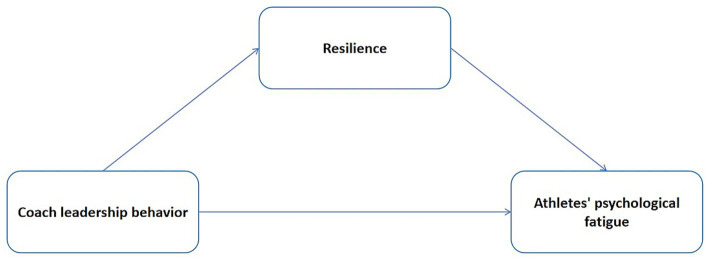
Schematic diagram of the research hypothesis model.

To date, international research has demonstrated a link between coaches’ leadership behaviors and athlete fatigue; however, the vast majority of empirical studies are rooted in Western competitive sports systems (Arthur et al., 2017; Ho and Smith, 2020; Smith and McGannon, 2018; Turnnidge and Cote, 2019; Zhang and Chen, 2019). Specifically in the context of China, which has a unique competitive sports system. Competitive sports in China operate within a distinct sociocultural framework characterized by collectivist values, high power distance within hierarchical relationships, and a state-led talent development model epitomized by the “national system” (Zheng et al., 2019). Under this system, coaches wield significant authority from the athletes’ early years, and the athletes’ socialization process is deeply shaped to emphasize respect for and obedience to coaching directives (Li and Li, 2021). This implies that the psychological mechanisms underlying coaching leadership behaviors may exhibit characteristics distinct from those in Western contexts. Consequently, research findings from different cultural backgrounds may struggle to capture the factors influencing psychological fatigue among Chinese athletes, which provides the rationale for this study (Figure 1).

To further understand the cultural contextual differences described above, it is necessary to examine the mechanisms linking coaching leadership behaviors and athletes’ psychological fatigue. Psychological resilience, as a psychological resource that enables positive adaptation under stressful conditions, is considered a key mediating variable connecting the two (Santana et al., 2023). On the one hand, coaches’ autonomy-supportive or controlling behaviors can shape athletes’ levels of psychological resilience in coping with adversity (Moljord et al., 2014); on the other hand, individuals with high psychological resilience are more likely to view high-intensity training as a challenge rather than a threat, thereby mitigating the onset of psychological fatigue (Varela et al., 2020). However, psychological resilience is not a decontextualized, universal construct; its developmental pathways and manifestations are deeply influenced by cultural values and social expectations (Zuo and Bai, 2025). Within the high-power-distance mentor-apprentice relationships in China, the development of athletes’ resilience may rely more heavily on the coach’s authoritative endorsement, suggesting that the effects of this mediating pathway in Chinese samples may differ from those in Western contexts. To date, research specifically examining the mediating effects of psychological resilience within this cultural context remains limited (Li, Li et al., 2025), providing a starting point for this study.

**Figure 2:**
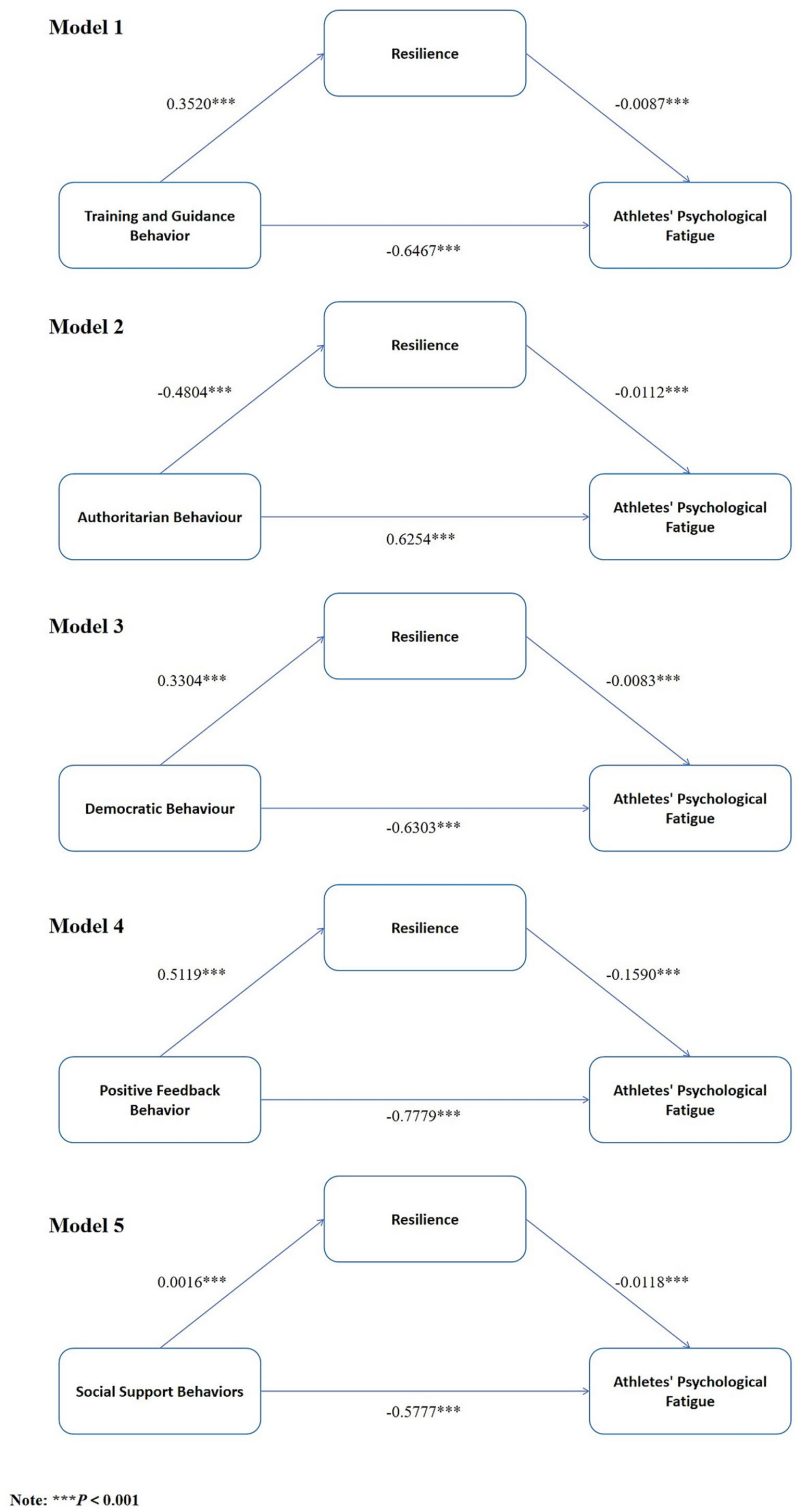
Mediational model diagram.

Based on the above analysis, this study selected Chinese basketball players as the sample to examine the mediating effect of psychological resilience on the relationship between coaches’ leadership behaviors and athletes’ psychological fatigue. By situating the research within the unique cultural and organizational context of China, this study not only seeks to validate and extend the cultural validity of existing theoretical models but also aims to reveal the localized psychological mechanisms underlying the development of psychological fatigue in sports. This will provide contextually relevant evidence for enhancing coaching practices and promoting athletes’ mental health in the Chinese context (Figure 2).

### 1.1 The relationship between coaches’ leadership behavior and athletes’ psychological fatigue

As the direct organizers and leaders of athletic training, coaches’ instructional behaviors profoundly influence athletes’ psychological states, including psychological states such as increased anxiety, decreased self-esteem, and emotional exhaustion (Arthur et al., 2017). Coaching behaviors typically encompass multiple dimensions, including democratic behaviors, authoritarian behaviors, training guidance behaviors, social support behaviors, and positive feedback behaviors (Turnnidge and Cote, 2019). These behaviors form the core social context of athletes’ daily training environments. Existing research has preliminarily revealed the association between coaches’ behaviors and athletes’ psychological fatigue. A survey by Liu involving 556 athletes found that coaches’ democratic leadership behaviors, training guidance behaviors, social support behaviors, and positive feedback behaviors were all significantly negatively correlated with athletes’ psychological fatigue, while authoritarian leadership behaviors were positively correlated with psychological fatigue (Liu et al., 2025). This implies that when coaches provide greater instructional support, emotional care, and positive feedback, athletes experience lower levels of psychological fatigue; conversely, unsupportive coaching styles may exacerbate athletes’ mental exhaustion. Additional research among boxers has revealed that coaching leadership behaviors mediate the influence of external environmental factors—such as social support—on athletes’ psychological fatigue (Zhang et al., 2023). In other words, coaches’ instructional behaviors not only directly influence athletes’ psychological fatigue but may also serve as a crucial contextual variable that mediates the effects of other factors on athletes’ psychological states. Based on this, the study proposes Hypothesis 1: Coaching leadership behaviors significantly predict athletes’ psychological fatigue. Specifically, democratic behavior, training guidance, social support, and positive feedback negatively predict psychological fatigue, while authoritarian behavior positively predicts it.

### 1.2 The relationship between coaching leadership practices and athletic psychological resilience

Psychological resilience is defined as an individual’s capacity to effectively cope with and adapt to stress, adversity, or trauma (Moljord et al., 2014). In the realm of competitive sports, it is regarded as a crucial psychological resource that shields athletes from negative psychological consequences (Garrido-Muñoz et al., 2024). Investigating how coaching behaviors influence athletes’ psychological resilience is crucial for understanding the psychological adaptation mechanisms of athletes, as it reveals the environmental antecedents of this vital stress-buffering resource. Social cognitive theory provides a foundational explanatory framework for this relationship. Central to this theory is the concept of triadic reciprocal determinism, where personal factors, behavior, and the environment continuously interact (Funasaki et al., 2025). In the context of sport, a coach’s leadership behavior constitutes a powerful environmental factor. When coaches consistently model adaptive coping strategies, provide mastery-oriented feedback, and create a supportive training climate, athletes can internalize these patterns through observational learning and direct reinforcement (Li et al., 2021). This social learning process gradually shapes athletes’ cognitive appraisals and behavioral responses to adversity, thereby fostering the development of greater psychological resilience (Varela et al., 2020). Conversely, a negative coaching environment may model maladaptive responses, hindering this development. Furthermore, Self-Determination Theory (SDT) posits that the extent to which coaches provide autonomy support, competence affirmation, and relational care directly influences the degree to which athletes’ fundamental psychological needs for autonomy, competence, and relatedness are satisfied (Deci and Ryan, 2017). When these needs are met, athletes are theorized to develop greater psychological resilience as a function of enhanced intrinsic motivation and more adaptive stress appraisals. Conversely, controlling coaching behaviors may thwart need satisfaction, thereby hindering the development of resilience (Jowett et al., 2017; Langan et al., 2015).

Previous research has demonstrated a link between coaching behavior and psychological resilience. Jooste‘s survey of athletes revealed that democratic coaching leadership styles showed a significant positive correlation with athletes’ psychological resilience, whereas authoritarian leadership styles exhibited a negative correlation with psychological resilience (Jooste and Kubayi, 2018). A study involving 301 volleyball players indicated that transformational coaching leadership significantly enhanced resilience by promoting task engagement and improving the coach-athlete relationship (Kao et al., 2025). Furthermore, research on paternalistic leadership has found that benevolent and virtue-based leadership positively predict athletes’ resilience (Liu et al., 2022). The aforementioned research indicates that supportive coaching behaviors may serve as a crucial environmental factor in cultivating athletes’ psychological resilience, with its mechanism involving multiple pathways such as need fulfillment, social learning, and the transmission of social support. Therefore, the study proposes Hypothesis 2: Coaching leadership behaviors significantly predict athletes’ psychological resilience. Specifically, democratic behavior, training guidance, social support, and positive feedback positively predict psychological resilience, while authoritarian behavior negatively predicts it.

### 1.3 The relationship between athletes’ psychological resilience and psychological fatigue

In the context of competitive sports, resilience is defined as a dynamic psychological asset that enables athletes to withstand and recover from the unique pressures of training and competition (Bryan et al., 2019; Fletcher and Sarkar, 2012). In contrast, mental fatigue is a psychophysiological state caused by prolonged cognitive activity, whose core characteristics include a subjective sense of exhaustion, depletion of energy, reduced motivation, and impaired cognitive performance (Chang et al., 2012; Van Cutsem et al., 2017). From a theoretical perspective, the role of resilience in mitigating psychological fatigue can be explained by the Sport Resilience Meta-Model, which posits that psychological resilience acts as a buffering mechanism during stressful situations (Gupta and McCarthy, 2022). According to this framework, when athletes face stressors on physical, cognitive, or psychosocial levels, their resilience resources influence how they evaluate these stressors and respond to them. Athletes with high levels of resilience tend to view stressful situations as challenges rather than threats, thereby activating more adaptive coping strategies and effectively conserving mental energy (Mei et al., 2025). Conversely, when resilience is insufficient to meet the demands of a given situation, an athlete’s psychological resources will gradually be depleted, ultimately manifesting as fatigue or burnout.

Complementing the stress response perspective is the explanation of the relationship between resilience and fatigue offered by cognitive resource theory (Yeo and Yap, 2023). In competitive sports, high-cognitive-demand activities such as tactical decision-making and attention control continuously deplete limited cognitive resources (Lee et al., 2017). Psychological resilience is believed to enhance the stability of cognitive function under stressful conditions, thereby reducing the rate at which cognitive resources are depleted (Chang et al., 2018; Shang and Yang, 2021). In other words, athletes with high levels of resilience may be better able to focus on their performance and are less likely to experience subjective feelings of mental fatigue. This assertion is also supported by empirical research. A systematic review involving 3,302 athletes indicated that psychological resilience influences the onset of burnout through two primary pathways: individual factors (such as stress perception, coping strategies, and motivational orientation) and environmental factors (such as social support, motivational climate, family cohesion, and the quality of the coach-athlete relationship). The results confirmed that psychological resilience has a clear protective effect against burnout and mental fatigue in athletes (Cai et al., 2025). Furthermore, research has further demonstrated that there is a significant negative correlation between psychological resilience and burnout symptoms among adolescent athletes (Santana et al., 2023). Studies of elite athletes have similarly confirmed that psychological resilience serves as a protective factor in reducing the risk of burnout. More importantly, the longitudinal study by Sorkkila provides evidence that psychological resilience is not only associated with current fatigue levels but also prospectively predicts reduced future burnout and dropout behavior among student athletes, suggesting the protective nature of psychological resilience over time (Sorkkila et al., 2019). In summary, both theoretical and empirical findings point to the possibility that psychological resilience may serve as a key factor in buffering the development of psychological fatigue in athletes. Therefore, based on the above evidence, this study proposes Hypothesis 3: Athletes’ psychological resilience significantly and negatively predicts their psychological fatigue.

### 1.4 The mediating model proposed by the study

Building upon the theoretical and empirical evidence presented above, this study proposes a mediation model in which athletes’ psychological resilience serves as a key mediating mechanism linking coaching leadership behaviors to psychological fatigue. To provide a coherent theoretical foundation for this model, we draw upon the Conservation of Resources (COR) theory (Hobfoll, 1989). COR theory posits that individuals strive to obtain, retain, protect, and foster resources that they value. Resources include personal characteristics (e.g., resilience, self-efficacy), conditions (e.g., supportive coach-athlete relationship), and energies (e.g., physical and emotional well-being). According to COR theory, resource loss is disproportionately more salient than resource gain, and stress occurs when individuals face threat of resource loss, actual resource loss, or failure to gain resources after significant investment (Hobfoll, 2001). Within the sport context, coaching leadership behaviors can be conceptualized as environmental conditions that either facilitate or hinder athletes’ resource acquisition and preservation. Specifically, positive, supportive, and democratic coaching behaviors (e.g., providing constructive feedback, showing respect, involving athletes in decision-making) are likely to enhance athletes’ psychological resilience by fostering a resource-rich environment. Such behaviors help athletes develop coping skills, self-efficacy, and social support networks—all of which are key components of psychological resilience (Fletcher and Sarkar, 2012). In contrast, autocratic, controlling, or negative coaching behaviors may deplete athletes’ existing resources, undermining their capacity to bounce back from setbacks. When athletes possess higher levels of psychological resilience—a personal resource—they are better equipped to manage stressors, regulate emotions, and maintain motivation despite challenges. This resource, in turn, serves as a protective buffer against the development of psychological fatigue, which is characterized by emotional and physical exhaustion, reduced sense of accomplishment, and sport devaluation (Raedeke and Smith, 2001). According to COR theory, resilient athletes are less likely to experience a loss spiral (i.e., initial resource loss begets further loss) because they can mobilize alternative resources to offset potential threats.

Therefore, based on the aforementioned theoretical model and research evidence, this study proposes the following hypotheses:

*H1:* Coaching leadership behaviors significantly predict athletes’ psychological fatigue. Specifically, democratic behavior, training guidance, social support, and positive feedback negatively predict psychological fatigue, while authoritarian behavior positively predicts it.

*H2:* Coaching leadership behaviors significantly predict athletes’ psychological resilience. Specifically, democratic behavior, training guidance, social support, and positive feedback positively predict psychological resilience, while authoritarian behavior negatively predicts it.

*H3:* Athletes’ psychological resilience significantly and negatively predicts their psychological fatigue.

*H4:* Athletes’ psychological resilience mediates the relationship between the coach’s leadership behavior and psychological fatigue.

## 2 Methods

### 2.1 Participants and testing methods

This study used G*Power 3.1 software to calculate the required sample size (Faul et al., 2007). A moderate effect size of f^2^ = 0.15, a significance level of *α* = 0.05, and a statistical power of Power (1-β) = 0.95 were set (Schoemann et al., 2017). The results indicated that the minimum required sample size was 300 participants. Therefore, from August to December 2025, basketball players were recruited via convenience sampling through an online questionnaire from sports management centers in Shandong, Sichuan, Guangdong, Zhejiang, and Liaoning provinces. These provinces represent key hubs for basketball development in China, all ranking among the top eight regions in the National Games basketball events. This approach aimed to maximize sample representativeness. A total of 450 questionnaires were distributed, with 408 returned. After excluding 76 invalid or non-compliant responses, 332 valid questionnaires were ultimately included in the final analysis. The demographic and athlete characteristics of the participant sample are summarized in Table 1. This study strictly adheres to the ethical guidelines established by the 1964 Declaration of Helsinki and its subsequent revisions, which constitute a widely recognised set of ethical principles for research involving human subjects and have been universally adopted in the fields of social and behavioral sciences. We confirm that all methods employed in this study have been approved by the Ethics Committee of the School of Physical Education at Qufu Normal University. Furthermore, we confirm that informed consent has been obtained from all participants (approval number: QF2006003).

**Table 1:** Characteristics of the athletes (*N* = 332).

Variable		*N*	Percentage
Gender	Male	210	63.25%
	Female	122	36.75%
Age group	18–21	203	61.14%
	21–24	73	21.99%
	25–28	32	9.64%
	≥29	24	7.23%
Sports level	Second-class athlete	231	69.58%
	First-class athlete	94	28.31%
	Elite-level athlete	8	2.41%
Years of training	1–5 years	89	26.81%
	6–10 years	152	45.78%
	11–14 years	72	21.69%
	>15 years	9	2.71%
Family type	Complete family	287	86.45%
	Single-parent family	22	6.63%
	Blended family	23	6.93%
Type of household egistration	Urban	238	71.69%
	Rural	94	28.31%

Basketball is one of the most popular sports in China. According to the China Basketball Development Report released by the Chinese Basketball Association in 2021, the country has approximately 125 million general basketball participants and 76.1 million core basketball participants, making basketball the most participated team sport in China (Chinese Basketball Association, 2021). It is estimated that over 30 million registered participants are involved in various levels of school and provincial teams, underscoring the sport‘s profound grassroots foundation and cultural significance in the Chinese context (Chinese Basketball Association, 2021). Secondly, basketball is a high-intensity, highly interactive team sport in which athletes frequently face complex tactical decisions and high cognitive demands during training and competition (Moreira et al., 2018; Silvestri et al., 2023). This makes psychological fatigue a particularly prominent issue. Furthermore, the quality of the relationship between coaches and athletes is considered one of the key factors influencing athletic performance, as it reflects the degree of interdependence between coaches and athletes on emotional, cognitive, and behavioral levels (Jowett et al., 2017; Jowett and Poczwardowski, 2007). This deliberate focus aims to enhance the internal validity of the research findings within this specific competitive context.

As shown in Table 1, male athletes account for a higher proportion (63.25%) of the sample. In terms of age distribution, the sample is primarily concentrated among young athletes aged 18 to 21 (61.14%), which aligns with the predominant age group in Chinese university and provincial sports teams. In terms of athletic ranking, Level 2 athletes constitute the vast majority (69.58%), while Level 1 and higher athletes account for approximately 30%. In China’s competitive sports system, according to the classification standards of the General Administration of Sport of China, “Elite-level athlete” typically refers to athletes who have achieved outstanding results in international or national competitions, “Level 1 athletes” refer to athletes who have achieved outstanding results in national or provincial championships, while “Level 2 athletes” typically refer to athletes who have performed exceptionally well in provincial competitions. Regarding years of training, the highest proportion of athletes (45.78%) has 6 to 10 years of systematic training experience. Overall, this sample reflects the characteristics of competitive athletes who are currently in a critical phase of both specialized skill development and physical and mental growth. Given that athletes at this stage face high-intensity training pressures and are highly sensitive to coaches’ leadership behaviors, exploring the relationship among leadership behaviors, psychological resilience, and psychological fatigue within this sample group holds significant practical importance.

### 2.2 Test scales

#### 2.2.1 Coach leadership behavior scale

The English version of the Sports Leadership Scale developed by Chelladurai (1984) was adopted and subsequently revised by Cai Duanwei into the Chinese version titled “Coach Leadership Behavior Scale (Cai, 2016).” This scale is used to measure athletes’ perceptions of coaching leadership behaviors. It consists of 31 items covering five dimensions: training guidance, democratic behavior, authoritarian behavior, social support, and positive feedback. For example, “When I perform well, my coach gives me positive feedback” (referring to training and coaching behaviors), and “When I make a mistake, my coach expresses dissatisfaction” (referring to authoritarian behaviors). The scale uses a 5-point Likert scale, ranging from 1 “Never” to 5 “Always.” Higher scores on each dimension indicate that athletes perceive the corresponding coaching leadership behavior more frequently. The scale does not include reverse-scored items. In the sample of this study, the Cronbach’s alpha coefficients for each subscale ranged from 0.898 to 0.941. Confirmatory factor analysis results showed that the five-factor structure possessed good construct validity (χ^2^/df = 1.356, CFI = 0.963, TLI = 0.976, RMSEA = 0.031, RMR = 0.076), indicating that the scale possesses good psychometric properties in the study sample.

#### 2.2.2 Athlete psychological fatigue scale

The study used the Physical and Mental Fatigue Scale developed by Zhang Liwei to assess psychological fatigue (Zhang, 2010). The scale consists of 15 items and includes three dimensions: reduced sense of accomplishment (e.g., “I do not feel a sense of accomplishment when exercising”), emotional/physical exhaustion (e.g., “Training leaves me feeling completely exhausted”), and negative evaluations of exercise (e.g., “I am becoming less and less concerned about my performance during exercise”). The scale uses a 5-point Likert scale, ranging from 1 (“Never”) to 5 (“Always”). Higher total scores and scores on each dimension indicate a more severe degree of psychological fatigue in athletes. The scale contains some reverse-scored items (primarily within the sense of accomplishment dimension), and these items were reverse-scored prior to data analysis. In the sample of this study, the reliability coefficients for each dimension ranged from 0.863 to 0.884. The results of confirmatory factor analysis indicated a good model fit (χ^2^/df = 1.323, CFI = 0.982, TLI = 0.989, RMSEA = 0.023, RMR = 0.037), suggesting that the scale possesses good reliability and validity in this sample.

#### 2.2.3 Sports psychological resilience scale

This study employed the Sport Mental Toughness Questionnaire (SMTQ) developed by Sheard et al. (2009), which was subsequently translated into Chinese by Wang et al. (2014). The questionnaire consists of 14 items across three dimensions—self-confidence, stability, and sense of control—with four items in each dimension. A sample item is, ‘I can quickly adjust my mindset and refocus after making a critical mistake during a game.’The questionnaire uses a 5-point Likert scale, ranging from 1 (“strongly disagree”) to 5 (“strongly agree”). Higher scores indicate a higher level of psychological resilience among athletes. All items in the questionnaire are positively scored; there are no reverse-scored items. In the sample of this study, the Cronbach’s alpha coefficients for each dimension ranged from 0.813 to 0.842. The results of confirmatory factor analysis supported the three-factor structure of the questionnaire (χ^2^/df = 1.128, CFI = 0.946, TLI = 0.951, RMSEA = 0.041, RMR = 0.036).

### 2.3 Statistical analysis

All questionnaire data were preliminarily organized and coded using Excel 2021 (Microsoft Corporation, 2021), followed by statistical analysis with SPSS 26.0 (IBM, 2019) and AMOS 24.0 (Arbuckle, 2016). First, descriptive statistical analyses were conducted on each research variable to calculate metrics such as the mean and standard deviation, thereby presenting the central tendency and dispersion of the variables. Additionally, to assess the validity of the data distribution, tests for skewness and kurtosis were performed. If the absolute value of skewness is less than 2 and the absolute value of kurtosis is less than 7, this indicates that the data do not deviate significantly from a univariate normal distribution (Hoyle, 1995). Second, given that the data in this study are derived entirely from participants’ self-reports, we employed Harman’s one-way test to examine potential common-method bias. All measurement items were included in an exploratory factor analysis, and factors were extracted without rotation. If the first factor explains less than 40% of the total variance, this indicates that there is no serious common-method bias In addition, Pearson correlation analysis was used to examine the relationships among coaches’ leadership behaviors, psychological resilience, and athletes’ psychological fatigue. Third, a confirmatory factor analysis model was constructed in AMOS 24.0. Distinctive validity among variables was assessed by comparing model fit indices between the baseline model and alternative models. Model fit evaluation employed the following indices: Chi-square/degrees of freedom (χ^2^/df), Comparative Fit Index (CFI), Tucker-Lewis Index (TLI), Root Mean Square Error of Approximation (RMSEA), and Standardized Residual Mean Square Root (SRMR). A χ^2^/df < 3, CFI and TLI > 0.90, and RMSEA and SRMR < 0.08 indicated good model fit (Kline, 2016). Finally, to examine the mediating role of psychological resilience between coaching guidance behaviors and athletes’ psychological fatigue, a structural equation model was constructed in AMOS 24.0. The model included coaches’ leadership behavior as an exogenous latent variable (with its five dimensions as observed indicators), psychological resilience as the mediating variable, athlete psychological fatigue as the outcome variable, and incorporated gender, years of training, and athletic level as control variables. The significance of the mediating effect was tested using bias-corrected bootstrap sampling (5,000 repeated samples). If the 95% confidence interval for the indirect effect did not include zero, the mediating effect was considered statistically significant (Preacher and Hayes, 2008).

## 3 Results

### 3.1 Common method Bias

To minimise potential common-method bias, anonymous survey administration and reverse-scored items were employed during data collection, supplemented by Harman’s one-way test (Podsakoff et al., 2003). This method is a widely used diagnostic tool for assessing common-method bias in survey research, and has also been adopted in recent studies on athlete burnout and coaching behavior (Healy et al., 2014; Hu et al., 2023). The results indicated that 14 factors had eigenvalues greater than 1. The first factor explained 33.46% of the variance, which is below the critical threshold of 40% (Tang and Wen, 2020), suggesting that there was no serious common-method bias in this study.

### 3.2 Correlation analysis

As shown in Table 2, coaching leadership behaviors exhibited a significant negative correlation with athletes’ psychological fatigue (*p* < 0.01). Among these, authoritarian behavior showed a positive correlation with psychological fatigue (*p* < 0.01), while all other dimensions demonstrated negative correlations. Athlete psychological resilience exhibited a significant negative correlation with athlete psychological fatigue (*p* < 0.01). This indicates that the aforementioned variables are significantly correlated. Psychological resilience may exert a mediating influence in the relationship between coaching leadership behaviors and athlete psychological fatigue, providing a basis for subsequent testing of this mediating effect.

**Table 2:** Descriptive statistics and correlation analysis of variables (*N* = 332).

Variable	*M*	SD	1	1.1	1.2	1.3	1.4	1.5	2	3
1. Coaching leadership behavior	3.48	0.70	1							
1.1 Training and guidance practices	3.70	0.92	0.942^**^	1						
1.2 Authoritarian conduct	3.51	0.75	0.930^**^	0.842^**^	1					
1.3 Democratic practices	3.76	0.96	0.944^**^	0.903^**^	0.850^**^	1				
1.4 Positive feedback behavior	2.79	0.59	0.324^**^	0.125^**^	0.275^**^	0.104^**^	1			
1.5 Social support	3.40	0.73	0.907^**^	0.813^**^	0.805^**^	0.825^**^	0.292^**^	1		
2 Resilience	3.08	0.52	0.746^**^	0.627^**^	0.698^**^	0.615^**^	0.584^**^	0.689^**^	1	
3 Athletes’ psychological fatigue	2.52	0.72	−0.156^**^	−0.315^**^	0.127^*^	−0.140^**^	−0.185^**^	−0.104^**^	−0.296^**^	1

^*^p < 0.05; ^**^p < 0.01.

### 3.3 Regression analysis

To further investigate the complex relationship among coaches’ leadership behaviors, athletes’ psychological fatigue, and psychological resilience, two distinct regression models were employed for regression analysis. Age, years of training, and competition level were included as control variables.

#### 3.3.1 Regression analysis of coaching leadership behaviors on athletes’ psychological fatigue

A regression model was constructed with five dimensions of coaching leadership behaviors as independent variables and athletes’ psychological fatigue as the dependent variable. As shown in Table 3, training guidance, democratic behavior, positive feedback, and social support significantly predicted athletes’ psychological fatigue negatively, while authoritarian behavior significantly predicted it positively.

**Table 3:** Regression analysis of coaches’ leadership behaviors on athletes’ psychological fatigue (*N* = 332).

Related variables and coefficient	Non-standardization coefficient		Standardized coefficient	*t*	*p*	Collinearity diagnosis	
	*β*	S.E	Beta			VIF	Tolerance
Total	−1.17	0.143		−8.172	0.000^***^		
1.1 Training and guidance practices	−0.205	0.064	−0.262	−3.184	0.002^**^	1.372	0.157
1.2 Authoritarian conduct	0.298	0.066	0.397	4.542	0.000^***^	1.225	0.138
1.3 Democratic practices	−0.143	0.068	−0.149	−2.096	0.037^*^	1.772	0.21
1.4 Positive feedback behavior	−0.115	0.065	−0.117	−1.784	0.005^**^	1.039	0.248
1.5 Social support	−0.837	0.045	−0.684	−1.632	0.000^***^	1.271	0.787
R^2^	0.655						
Adjusted R^2^	0.649						
F	123.543^***^						
D-W value	1.603						

^*^p < 0.05; ^**^p < 0.01; ^***^p < 0.001.

#### 3.3.2 Regression analysis of coaching leadership behaviors on athletes’ psychological resilience

Regression analysis was conducted with the five dimensions of coaching leadership behaviors as independent variables and psychological resilience as the dependent variable. The results, as shown in Table 4, indicate that coaching guidance, democratic behavior, positive feedback, and social support significantly and positively predict athletes’ psychological resilience. Conversely, authoritarian behavior negatively predicts athletes’ psychological resilience.

**Table 4:** Regression analysis of coaching leadership behaviors on resilience (*N* = 332).

Related variables and coefficient	Non-standardization Coefficient		Standardized coefficient	*t*	*p*	Collinearity diagnosis	
	*β*	S.E	Beta			VIF	Tolerance
Total	0.523	0.097		5.4	0.000^***^		
1.1 Training and guidance practices	0.088	0.044	0.157	2.026	0.044^*^	1.372	0.157
1.2 Authoritarian conduct	−0.049	−0.044	−0.091	−1.103	0.001^**^	1.225	0.138
1.3 Democratic practices	0.155	0.046	0.225	3.35	0.001^**^	1.772	0.21
1.4 Positive feedback behavior	0.123	0.044	0.175	2.828	0.005^**^	1.039	0.248
1.5 Social support	0.387	0.03	0.442	2.741	0.000^***^	1.271	0.787
R^2^	0.691						
Adjusted R^2^	0.686						
F	145.843^***^						
D-W value	2.091						

^*^p < 0.05; ^**^p < 0.01; ^***^p < 0.001.

### 3.4 Testing for mediating effects

A Bootstrap mediation analysis was conducted to test the significance of the indirect effect, using Model 4 from the Process 4.0 SPSS macro developed by Hayes (2013). The five dimensions of coaching leadership behavior, athletes’ psychological resilience, and athletes’ psychological fatigue were treated as dependent variables, while controlling for gender, years of training, and skill level. A bootstrap method with 5,000 resamples was employed. All variables were standardized prior to analysis to enhance data interpretability and comparability. The five dimensions of coaching leadership behavior served as independent variables, athletic psychological fatigue as the dependent variable, and athlete psychological resilience as the mediating variable. The results of the mediation analysis, presented in Table 5, report the direct, indirect, and total effects for each leadership dimension. Findings indicate that the bootstrap confidence intervals for the indirect effects all excluded zero, confirming that psychological resilience’s mediating role was statistically significant across all models.

**Table 5:** Mediating role of resilience.

Related variables and coefficient	Mediation model	Effect categories	Effect value	Total effect proportion	Standard deviation	Bootstrap lower	Bootstrap upper
Model 1	Training and coaching practices → Resilience → Athlete psychological fatigue	Direct effect	−0.2469	38.18%	0.0393	−0.3275	−0.1662
		Indirect effects	−0.3998	61.82%	0.0620	−0.5190	−0.2784
		Total effect	−0.6467		0.0410	−0.7240	−0.5694
Model 2	Authoritarian behavior → Resilience → Athlete psychological fatigue	Direct effect	0.1221	19.52%	0.0525	0.0187	0.2254
		Indirect effects	0.5033	80.48%	0.0824	0.3387	0.6619
		Total effect	0.6254		0.0618	0.5038	0.7470
Model 3	Democratic behavior → Resilience → Athlete psychological fatigue	Direct effect	−0.2552	40.49%	0.0389	−0.3317	−0.1787
		Indirect effects	−0.3751	59.51%	0.0581	−0.2603	−0.4897
		Total effect	−0.6303		0.0362	−0.7015	−0.5591
Model 4	Positive feedback behavior → Resilience → Athlete psychological fatigue	Direct effect	−0.1138	11.95%	0.0335	−0.1807	−0.0491
		Indirect effects	−0.8388	88.05%	0.0491	−0.9354	−0.7422
		Total effect	−0.9526		0.0597	−1.0699	−0.8352
Model 5	Social support → Resilience → Athlete psychological fatigue	Direct effect	−0.1029	17.81%	0.0540	−0.2091	−0.0033
		Indirect effects	−0.4748	82.19%	0.0855	−0.3039	−0.6362
		Total effect	−0.5777		0.0542	−0.7040	−0.4515

## 4 Discussion

### 4.1 Relationship between coaching leadership behaviors and athletes’ psychological fatigue

The primary aim of the present study was to examine the relationship between coaching leadership behaviors and athletes’ psychological fatigue, with a particular focus on the mediating role of psychological resilience. The results of this study confirm that the various dimensions of coaching leadership behavior have a significant impact on athletes’ psychological fatigue, supporting Hypothesis H1. Unlike the preliminary findings based solely on correlation analysis, regression analysis further revealed the independent predictive effects of each dimension: after controlling for demographic variables, directive behavior (*β* = −0.262, *p* < 0.01), democratic behavior (*β* = −0.149, *p* < 0.05), positive feedback (*β* = −0.117, *p* < 0.01), and social support (*β* = −0.684, *p* < 0.001) all significantly and negatively predicted psychological fatigue, whereas authoritarian behavior exhibited a significant positive predictive effect (*β* = 0.397, *p* < 0.001). Notably, the negative predictive effect of social support was stronger, suggesting that emotional support provided by coaches may be the most powerful protective factor against psychological fatigue among Chinese basketball players. These regression model findings are consistent with previous research, which similarly identified authoritarian leadership as a risk factor for psychological fatigue and supportive leadership as a protective factor (Liu et al., 2025; Zhang et al., 2023). In basketball, coaches serve as the direct organizers and leaders of athletic training, exerting profound influence over athletes’ daily training routines, competition experiences, and psychological states (Bormann and Rowold, 2016; Zhang et al., 2025). Positive leadership behaviors—such as providing emotional support, granting autonomy, offering technical guidance, and delivering constructive feedback—can fulfill athletes’ fundamental psychological needs. These practices help athletes better manage high cognitive demands during training and competition, thereby reducing susceptibility to mental fatigue (Li, Cui et al., 2025).

Conversely, authoritarian leadership styles may exacerbate mental resource depletion by increasing athletes’ psychological pressure and cognitive load, thereby inducing or intensifying mental fatigue. From the perspective of SDT, authoritarian leadership behaviors by coaches—such as unilateral decision-making and rigid control—systematically hinder the fulfillment of athletes’ basic psychological needs (Deci and Ryan, 2017). Specifically, authoritarian behavior undermines athletes’ autonomy (lack of choice and voice), sense of competence (emphasis on error correction rather than mastery-oriented feedback), and sense of belonging (creating hierarchical distance rather than collaborative relationships) (Bartholomew et al., 2011). This multidimensional obstruction of needs forces athletes to expend significant cognitive and emotional resources on regulating negative emotions and maintaining superficial compliance, thereby accelerating the depletion of psychological energy, which manifests as increased levels of mental fatigue (Liu et al., 2025). In other words, reducing mental fatigue is crucial for maintaining athletes’ cognitive function, optimizing technical performance, and sustaining long-term commitment to sports.

Beyond these general mechanisms, the cultural context of competitive sports in China adds a unique dimension to this relationship. In a culture characterized by high power distance, coaches’ authority is often taken for granted, and authoritarian behavior may even be tacitly accepted as a necessary means to achieve the adage that “strict teachers produce outstanding students.” However, as a new generation of athletes develops a stronger sense of self-worth and greater concern for mental health, the tension between internalized traditional norms and the need for personal autonomy may exacerbate the psychological toll of authoritarian behavior. This cultural and psychological contradiction may well be a key contextual factor underlying the consistent positive correlation between authoritarian behavior and fatigue observed in this sample.

### 4.2 The relationship between coaching leadership behavior and psychological resilience

This study confirms that coaches’ leadership behaviors significantly influence athletes’ psychological resilience, supporting Hypothesis H2. Regression analysis (see Table 4) shows that coaching guidance (*β* = 0.157, *p* < 0.05), democratic behavior (*β* = 0.225, *p* < 0.01), positive feedback (*β* = 0.175, *p* < 0.01), and social support (*β* = 0.442, *p* < 0.001) all significantly and positively predicted psychological resilience, whereas authoritarian behavior significantly and negatively predicted psychological resilience (*β* = −0.091, *p* < 0.01). Among these, social support had the strongest predictive effect, further confirming the central role of emotional support in fostering psychological resilience in athletes. These findings are consistent with the patterns reported in prior research, where democratic leadership was positively associated with resilience, and authoritarian leadership was negatively associated with resilience, in diverse athlete populations including South African wheelchair basketball players and Chinese adolescent athletes (Jooste and Kubayi, 2018; Li et al., 2021).

Coaches’ leadership behaviors constitute the primary social context of athletes’ daily training, shaping their psychological traits through social learning and need-satisfaction mechanisms (Funasaki et al., 2025). When coaches exhibit supportive and democratic coaching behaviors, athletes gradually internalize the coaches’ coping strategies and value systems through observational learning and behavioral imitation, thereby enhancing their psychological resilience (Varela et al., 2020; Zhang et al., 2023). Specifically, supportive behaviors provide athletes with emotional support and a sense of belonging, enabling them to access external resources when facing pressure and adversity; coaching behaviors help athletes master skills and strategies for coping with challenges, thereby enhancing their self-efficacy; positive feedback reinforces athletes’ sense of competence, making them more likely to view difficulties as opportunities for growth rather than threats; and democratic behaviors grant athletes autonomy, fulfilling their need for self-determination, which in turn stimulates intrinsic motivation and persistence (Li et al., 2025; Walter et al., 2025; Yildirim et al., 2024). Conversely, authoritarian leadership styles hinder the fulfillment of athletes’ basic psychological needs through excessive control and authoritarian pressure, undermining their autonomy and sense of competence, and thus negatively impacting the development of psychological resilience (Donnelly et al., 2024; Jowett et al., 2017). The fulfillment of psychological needs is a key foundation for the development of psychological resilience, and coaches’ supportive leadership behaviors serve as important contextual factors in meeting these needs. Enhancing athletes’ psychological resilience plays a crucial protective role in strengthening their ability to cope with stress, resist mental fatigue, and maintain long-term participation in sports.

### 4.3 The relationship between psychological resilience and mental fatigue in athletes

This study also confirmed a significant negative correlation between athletes’ psychological resilience and psychological fatigue, supporting Hypothesis H3. This finding is consistent with the theoretical expectations of the stress coping process model, which posits that an individual’s psychological resources play a key buffering role in stress coping (Lu et al., 2016; Cai et al., 2025). When athletes possess higher levels of psychological resilience, they are better able to mobilize coping resources, perceiving high-cognitive-load situations in training and competition as “challenges” rather than “threats,” thereby slowing the rate of psychological resource depletion (Fletcher and Sarkar, 2012). Notably, psychological resilience not only directly reduces levels of mental fatigue but also serves as a protective buffer against daily stressors, suggesting a stable negative correlation between the two (Bryan et al., 2019). From the perspective of cognitive resource theory, athletes with high psychological resilience demonstrate greater attentional stability and emotional regulation when performing cognitively demanding tasks such as attentional control and tactical decision-making, thereby reducing the sense of fatigue caused by excessive depletion of cognitive resources (Lee et al., 2017; Yeo and Yap, 2023). This mechanism is consistent with previous findings regarding the role of psychological flexibility in buffering fatigue (Chang et al., 2018; Shang and Yang, 2021). Compared with existing research, these findings are highly consistent with the conclusions of a study on elite adolescent athletes, which found that psychological resilience is a significant negative predictor of fatigue symptoms (Sorkkila et al., 2019). However, the correlation coefficient between the two variables in this study was slightly lower than the effect size reported by Cai et al. (2025) in a group of elite athletes; this discrepancy may stem from differences in sample composition: the majority of participants in this study were second-tier athletes (69.58%), whose competitive level and long-term training stress differ from those of elite athletes. In other words, the buffering effect of psychological resilience against fatigue may be more pronounced among high-level athletes, suggesting that the resilience effect may exhibit a “threshold” characteristic, wherein its protective role is stronger under higher stress levels. Therefore, coaches and sports psychologists should consciously incorporate the cultivation of psychological resilience into daily intervention systems. By setting progressive challenge tasks, providing control-oriented feedback, and fostering a supportive team atmosphere, they can help athletes gradually accumulate experiences of successfully coping with stressful situations, thereby enhancing their psychological resilience resources.

### 4.4 The mediating role of psychological resilience

The mediation analysis indicates that psychological resilience mediates the relationship between coaching leadership behaviors and athletes’ psychological fatigue, supporting Hypothesis H4. This finding reveals the underlying mechanism through which coaching leadership behaviors influence athletes’ psychological fatigue: coaches’ leadership styles not only directly affect athletes’ levels of psychological fatigue but also exert an indirect effect by shaping athletes’ psychological resilience. Specifically, democratic behavior, social support behavior, training guidance behavior, and positive feedback behavior all indirectly reduce mental fatigue by enhancing psychological resilience; conversely, authoritarian behavior indirectly exacerbates mental fatigue by weakening psychological resilience, with a mediation effect proportion as high as 80.48%. Taking training guidance as an example, when coaches provide clear technical instructions and tactical advice, they not only directly help athletes cope with the cognitive load during training but also enhance their psychological resilience by boosting their sense of self-efficacy and competence (Langan et al., 2015; Maechel et al., 2020). Enhanced psychological resilience enables athletes to mobilize coping resources more effectively when facing cognitively demanding tasks, allowing them to perceive stressful situations as challenges rather than threats. This, in turn, slows the depletion of psychological resources and ultimately reduces psychological fatigue. Democratic coaching satisfies athletes’ basic psychological needs by fostering autonomy and involving them in decision-making, thereby strengthening intrinsic motivation and psychological resilience (Bates and McShan, 2025). Athletes with high psychological resilience maintain more stable emotional states and attention allocation when facing cognitive demands during training and competition, reducing unnecessary depletion of cognitive resources and thereby sustaining lower levels of mental fatigue. It is worth noting that even low-level, chronic mental fatigue can have insidious cumulative effects on athletes’ training quality and long-term athletic development (Russell et al., 2019). The ability of psychological resilience to mitigate this low-grade, persistent fatigue may therefore be just as practically significant as its role in buffering against acute, high-intensity exhaustion (Levillain et al., 2024; Zhang and Chen, 2025). Social support and positive feedback also exert their effects through the mediating pathway of psychological resilience: supportive interactions help athletes establish secure interpersonal relationships, enhancing their confidence and ability to cope with stress; positive feedback validates athletes’ efforts and achievements, reinforcing their sense of self-worth and persistence (Bormann and Rowold, 2016; Walter et al., 2025).

These supportive behaviors collectively promote the development of psychological resilience, and high psychological resilience, acting as a protective resource, effectively buffers the depletion of psychological resources caused by daily training loads. It is worth emphasizing that mediation analyses reveal that authoritarian behavior not only directly exacerbates fatigue but also exerts indirect negative effects by undermining psychological resilience (Takamatsu and Kawata, 2024). Unlike supportive behaviors, which build resilience through successful experiences and social buffering, authoritarian control appears to continuously deplete athletes’ psychological resources, making them more vulnerable to the effects of setbacks. Of course, the negative effects of authoritarian behavior do not manifest equally across all athletes or situations.

### 4.5 Theoretical implications

While our primary theoretical framework was the Conservation of Resources (COR) theory, it is also instructive to interpret the findings from the perspective of Self-Determination Theory (SDT; Deci and Ryan, 2017), although we did not directly measure its core constructs. From an SDT standpoint, coaching leadership behaviors that are autonomy-supportive (e.g., providing choice, rationale, and acknowledging athletes’ feelings) may satisfy athletes’ basic psychological needs for autonomy, competence, and relatedness (Vansteenkiste et al., 2020). The satisfaction of these needs, in turn, likely fosters psychological resilience and protects against psychological fatigue (Bartholomew et al., 2011). Conversely, controlling coaching behaviors could frustrate these needs, leading to resource depletion and increased fatigue (Kim et al., 2020). This post-hoc interpretation suggests that future research could directly examine the mediating role of basic psychological need satisfaction in the relationship between coaching behaviors and athlete mental health outcomes, thereby integrating COR and SDT perspectives. However, such an investigation would require validated measures of need satisfaction and frustration, which were beyond the scope of the current study.

### 4.6 Research limitations and future directions

The results of this study suggest that coaches can actively alleviate athletes’ mental fatigue through a potential mechanism—namely, fostering psychological resilience. This implies that interventions aimed at fostering psychological resilience may serve as a means to improve the quality of coach-athlete interactions and reduce athletes’ occupational burnout. Future longitudinal or experimental studies are needed to establish a causal relationship. In addition, this study has several other limitations. Firstly, although the SDT provides a guiding theoretical framework for the mediational model proposed in this study, it does not include direct measurements of its core constructs (such as the fulfilment or frustration of basic psychological needs). Consequently, we are unable to empirically test whether the influence of coaching behavior on psychological resilience is specifically mediated through these needs. Future research should incorporate these psychological need variables as mediating mechanisms to conduct a direct and comprehensive examination of the SDT-based pathways. Secondly, the inclusion of additional variables, such as training satisfaction and team efficacy, could provide a more comprehensive research perspective. Thirdly, there are certain limitations regarding the representativeness of the sample size. This study employed convenience sampling to survey basketball players in the relevant regions; the sample may not fully represent all basketball players nationwide across different backgrounds and skill levels. The use of convenience sampling may limit the generalisability of the findings to a broader population of basketball players. Fourthly, future research should investigate potential moderating variables—such as athletes’ trait resilience, growth mindset, or the quality of the coach-athlete relationship—that may buffer the harmful effects of authoritarian coaching behavior. Identifying such moderators could inform the development of coaching interventions that effectively balance the performance demands of competitive sport with the protection and promotion of athletes’ mental health. Consequently, it is recommended that future research consider including athletes from a wider range of sports, or conduct studies categorised by team versus individual sports, whilst further distinguishing differences in gender, sporting culture and sport-specific characteristics.

## 5 Conclusion

This study reveals the pathways through which coaching leadership behaviors influence athletes’ psychological fatigue and confirms the mediating role of psychological resilience. Specifically, the findings reveal a significant asymmetry: democratic, directive, supportive, and positive feedback behaviors alleviate fatigue by fostering psychological resilience, whereas authoritarian leadership behaviors exacerbate fatigue through a dual pathway—by hindering basic psychological needs and indirectly depleting psychological resources. These findings extend the application of SDT to competitive sports settings and highlight how the nature of coaching leadership behaviors influences athletes’ psychological energy.

Furthermore, the findings of this study offer actionable guidance for relevant stakeholders in sports settings. First, coach training programs should incorporate modules on communication that supports psychological needs to help coaches master specific strategies for enhancing—rather than depleting—athletes’ resilience when providing feedback, organizing training, and exercising authority. Second, sports governing bodies should include athletes’ mental health and sustainable development capabilities in the evaluation metrics for coaching effectiveness, rather than relying solely on competitive performance as the sole criterion. Third, sports psychologists can design relevant intervention programs based on the mediating role of psychological resilience—such as resilience training workshops and assessments of need-supportive team atmospheres—to mitigate the negative impact of inevitable coaching stressors on athletes.

## Data Availability

The original contributions presented in the study are included in the article/Supplementary material, further inquiries can be directed to the corresponding author/s.
